# Swine IRF3/IRF7 attenuates inflammatory responses through TLR4 signaling pathway

**DOI:** 10.18632/oncotarget.18740

**Published:** 2017-06-28

**Authors:** Pei-Ge Chen, Yan-Jing Guan, Guang-Ming Zha, Xian-Qin Jiao, He-Shui Zhu, Cheng-Yu Zhang, Yue-Ying Wang, He-Ping Li

**Affiliations:** ^1^ Key Laboratory of Animal Biochemistry and Nutrition, Ministry of Agriculture, Henan Agricultural University, Zhengzhou, Henan, China

**Keywords:** IRF3/IRF7, inflammatory response, overexpression, TLR4 signaling pathway, lipopolysaccharide

## Abstract

To explore the role of IRF3/IRF7 during inflammatory responses, we investigated the effects of swine IRF3/IRF7 on TLR4 signaling pathway and inflammatory factors expression in porcine kidney epithelial PK15 cell lines. We successfully constructed eukaryotic vectors PB-IRF3 and PB-IRF7, transfected these vectors into PK15 cells and observed GFP under a fluorescence microscope. In addition, RT-PCR was also used to detect transfection efficiency. We found that IRF3/IRF7 was efficiently overexpressed in PK15 cells. Moreover, we evaluated the effects of IRF3/IRF7 on the TLR4 signaling pathway and inflammatory factors by RT-PCR. Transfected cells were treated with lipopolysaccharide (LPS) alone, or in combination with a TBK1 inhibitor (LiCl). We revealed that IRF3/IRF7 enhanced IFNα production, and decreased IL-6 mRNA expression. Blocking the TBK1 pathway, inhibited the changes in IFNα, but not IL-6 mRNA. This illustrated that IRF3/IRF7 enhanced IFNα production through TLR4/TBK1 signaling pathway and played an anti-inflammatory role, while IRF3/IRF7 decreased IL-6 expression independent of the TBK1 pathway. Trends in MyD88, TRAF6, TBK1 and NFκB mRNA variation were similar in all treatments. LPS increased MyD88, TRAF6, TBK1 and NFκB mRNA abundance in PBR3/PBR7 and PBv cells, while LiCl blocked the LPS-mediated effects. The levels of these four factors in PBR3/PBR7 cells were higher than those in PBv. These results demonstrated that IRF3/IRF7 regulated the inflammatory response through the TLR4 signaling pathway. Overexpression of swine IRF3/IRF7 in PK15 cells induced type I interferons production, and attenuated inflammatory responses through TLR4 signaling pathway.

## INTRODUCTION

Outbreaks of catastrophic swine diseases have drawn extensive attention to the considerable economic and social losses that affect a wide range of swine farms locally and the industry worldwide. Current vaccination strategies and antiviral drugs cannot effectively control swine diseases, such as porcine reproductive and respiratory syndrome (PRRS), which is mainly caused by PRRS virus (PRRSV), based on PRRSV itself is easy to mutate with time, PRRSV has the diversity of the genotypes. The complex genetic diversity brings a challenge to increase the efficacy of current PRRS vaccines [1, 2]. The innate immune response is the first line of host defense against infections. Type I interferons (IFNs) (primarily IFN-α/β) are induced to boost the immune response, protecting hosts from viral and nonviral pathogens [3]. IFNs can inhibit a variety of viruses, bacteria, parasites, and bacterial and viral co-infections. Therefore, the role of IFNs has been focused on in the prevention and treatment of swine diseases in recent years. Initiation of IFN genes transcription depends on the timely recognition of pathogen-associated molecular patterns through host pattern recognition receptors within endosomes (such as Toll-like receptors, TLRs) or in the cytosol (including cytosolic RNA sensors, RNA helicases, and cytosolic DNA sensors) [4]. Recognition triggers distinct signaling pathways that converge on the activation of transcription factors called IFN regulatory factors (IRFs) [3], which include nine members (IRF-1 to 9). Among the IRF family, IRF1, IRF3, and IRF7 are characterized as acting early in regulating type I IFNs expression [3]. Moreover, IRF3 and IRF7 are two important members of IRFs family, having high homology. They play an important regulatory role in the expression and secretion of type I IFN for antiviral functions [5].

Moreover, IRF3 and IRF7 are primarily responsible for TLR4-IFN signaling through the TIR domain-containing adaptor protein pathway [6]. During *Lactobacillus acidophilus* infection, IRF1, IRF3, and IRF7 are involved in IFN-β responses in dendritic cells triggered by myeloid differentiation factors (MyD88) [7]. These results suggest that multiple IRF members often work together to coordinately regulate type I IFN gene activation, although the molecular mechanisms involved remain unclear. IRF1, IRF3, and IRF7 might simultaneously regulate IFN gene expression in certain tissues or cell types through similar signaling pathways, such as the MyD88 pathway [8]. Notably, IRF3 and IRF7 are necessary for the retinoic acid-inducible gene I-like receptors mediated IFN response [9], as well as for the MyD88-mediated IFN response [10]. Moreover, IFN suppression by viruses may be achieved through blocking IRF3/IRF7 activity [11]. IRF3/IRF7 undergo phosphorylation and enter the nucleus through nuclear localization signals, increasing IFN activity [12].

IRF3/IRF7 has been shown to regulate cell proliferation, apoptosis, inflammation, innate immune responses and insulin resistance [13–15]. Both can be activated by LPS, which exists mainly in the cell wall of Gram-negative bacteria. LPS binds to its receptor TLR4, located on the cell surface, activating TLR4-mediated signal transduction pathways, then activating downstream TRIF-dependent signal pathway, leading to IRF3/IRF7 phosphorylation, which regulates expression and secretion of type I IFNs.

Currently, studies on swine IRF3 and IRF7, especially regarding their function, are very few. To better understand IRF3/IRF7 function in preventing and resolving swine diseases, we constructed the eukaryotic expression vectors PB-IRF3 and PB-IRF7, and transfected them into PK15 cells to obtain IRF3/IRF7 overexpression. Meanwhile, we explored the effects of swine IRF3/IRF7 on TLR4 signaling pathway, using LPS alone, or in combination with a TBK1 inhibitor (LiCl). We found that overexpress IRF3/IRF7 in PK15 cells could activate TLR4 signal pathway through regulating MyD88, TBK1, NFκB and TRAF6 expression. Moreover, it promoted IFNα and inhibited IL-6 expression, further suppressing the inflammatory response. These results demonstrated that overexpression of swine IRF3/IRF7 could decrease the inflammatory response through TLR4 signaling pathway, and participate in type I interferons production.

## RESULTS

### Observation of transfection efficiency

**Figure 1:**
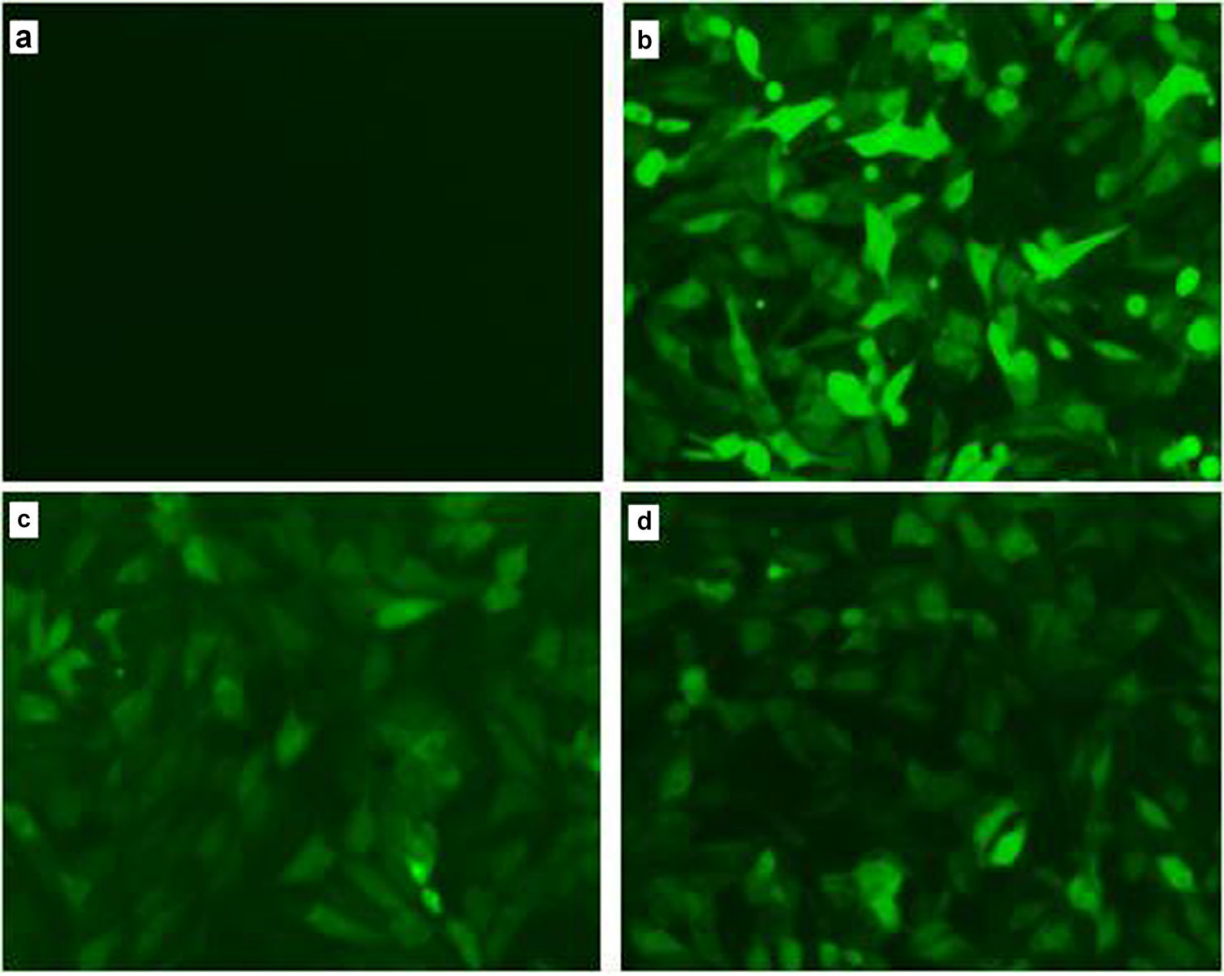
Observation of green fluorescence in PK15 cells transfected with IRF3/IRF7 expression vectors Green fluorescence was observed clearly using fluorescence microscopy in the PBv **(b)**, PBR3 **(c)** and PBF7 **(d)** cells, but not in non-transfected PK15 cells **(a)**.

The PB vector carries the GFP gene. When the PB vector is integrated into the host cell genome, GFP protein is expressed in the host cells, which can be seen using a fluorescence microscope. We clearly observed green fluorescence in the PBR3, PBF7, and PBv transfected, but not in the non-transfected, PK15 cells [Figure 1]. These results indicated that vectors with the GFP gene were successfully transfected into cells and integrated into the genome. Through screening, we obtained stable transfected cell lines.

### Overexpression of swine IRF3/IRF7 genes in PK15 cells

**Figure 2:**
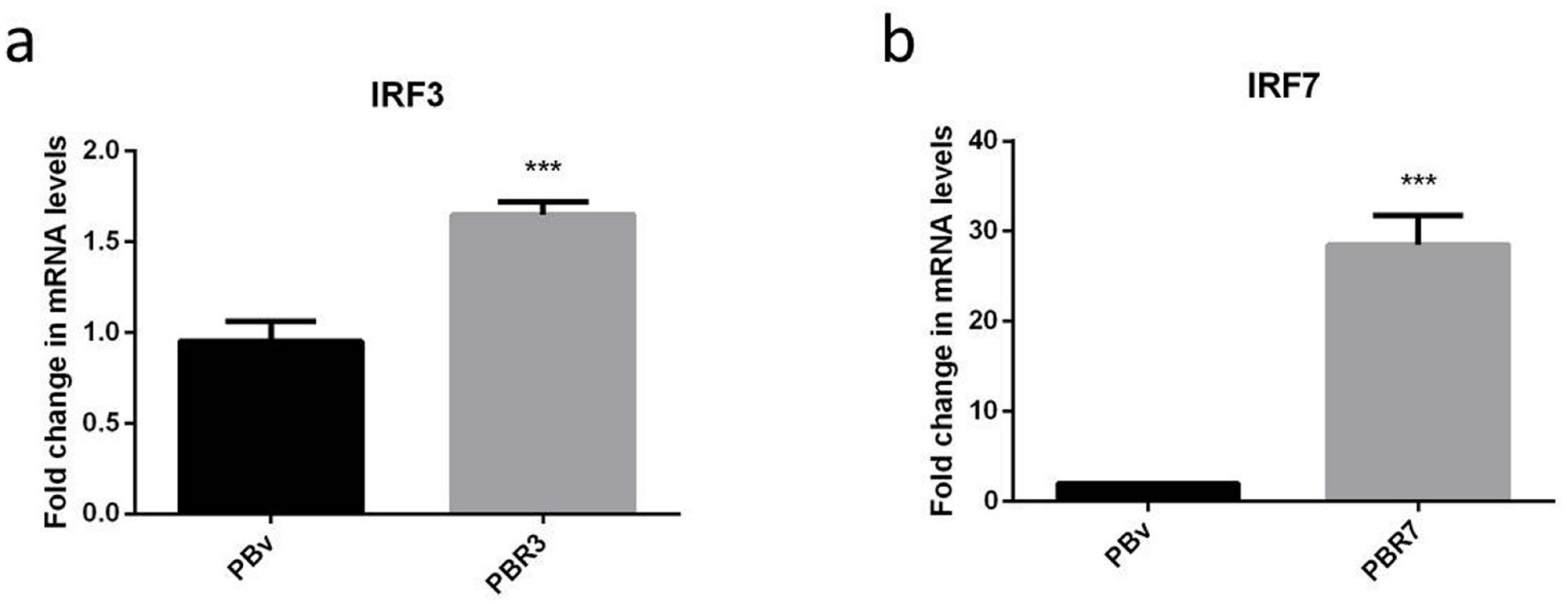
Overexpression of the swine IRF3/IRF7 gene in PK15 cells (**a and b)** demonstrate the overexpression of IRF3 and IRF7 mRNA in PBR3 and PBv and PBR7 and PBv cells, respectively. GAPDH was used as an internal housekeeping gene *** *P* < 0.001 vs. PBv.

We constructed the eukaryotic expression vectors PB-IRF3 and PB-IRF7, and transfected them into PK15 cells. To evaluate IRF expression efficiency, we detected the expression of IRF3/IRF7 using real-time RT-PCR. IRF3 and IRF7 mRNA expression were significantly higher in PBR3 and PBR7 cells than in PBv cells (*P*< 0.001) [Figure 2a, 2b]. Thus, IRF3/IRF7 was successfully transfected into PK15 cells and efficiently overexpressed.

### Swine IRF3/IRF7 genes induce an anti-inflammatory response

**Figure 3:**
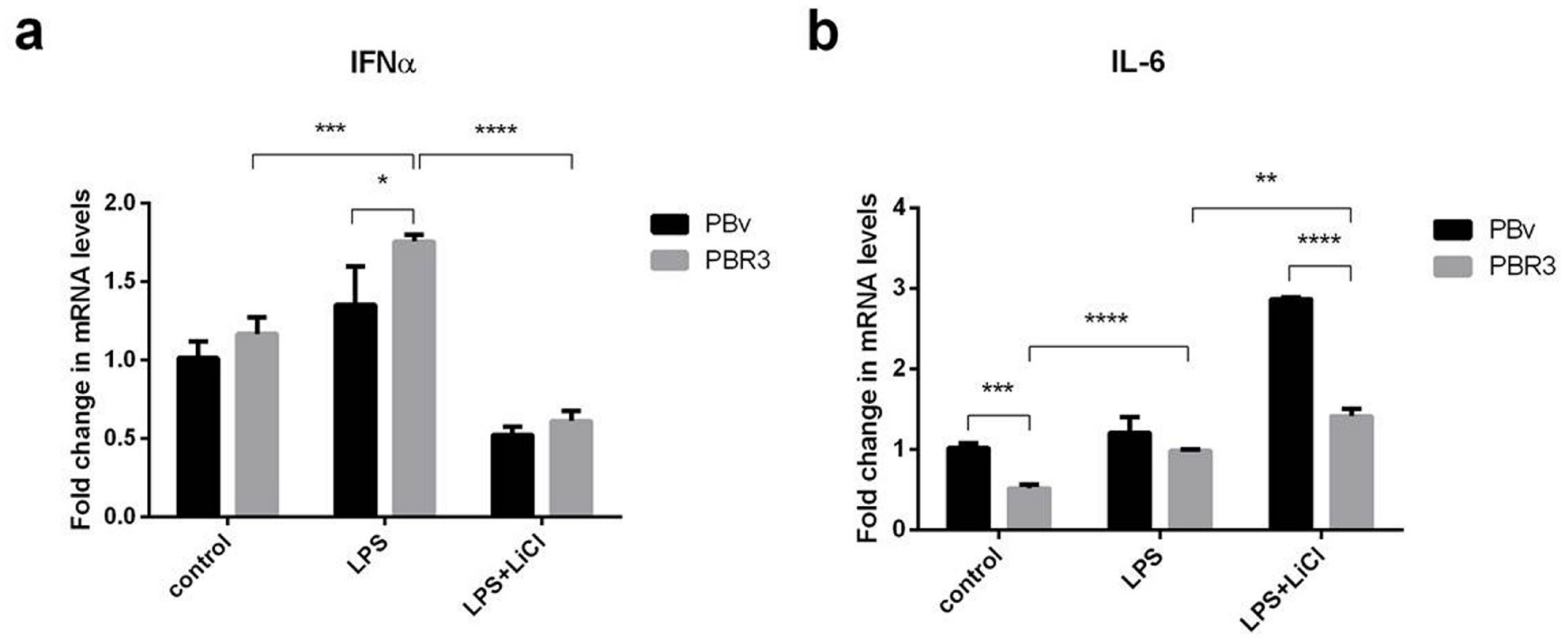
Effect of swine IRF3 on the expression of IFNα and IL-6 The expression of IFNα **(a)** and IL-6 **(b)** in PBR3 and PBv cells induced by LPS (50 μg/ml) with or without LiCl (20 mM) pretreatment. * *P* < 0.05, ** *P* < 0.01, *** *P* < 0.001 vs. PBv or between conditions.

**Figure 4:**
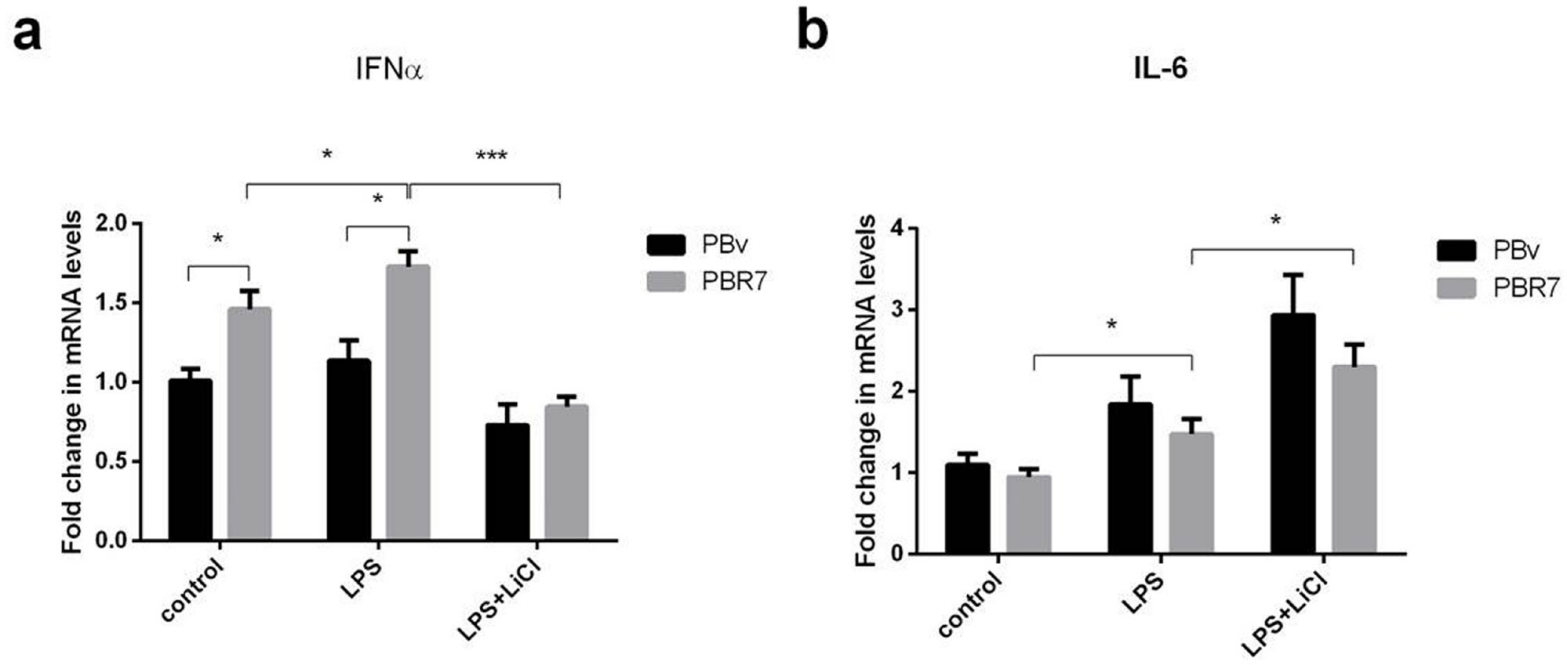
Effect of swine IRF7 on the expression of IFNα and IL-6 The expression of IFNα **(a)** and IL-6 **(b)** in PBR7 and PBv cells induced by LPS (50 μg/ml) with or without LiCl (20 mM) pretreatment. * *P* < 0.05, *** *P* < 0.001 vs. PBv or between conditions.

IRF3/IRF7 plays an important role in type I IFN production. Both exist in multiple types of cells, mainly in the cytoplasm in an inactive form. If cells are stimulated, IRF3/IRF7 are phosphorylated and change conformation, translocating to the nucleus. First, we explored the effect of swine IRF3/IRF7 overexpression on IFNα and IL-6 production. As shown in Figure 3a and Figure 4a, compared with control cells, LPS increased IFNα mRNA abundance in PBR3/PBR7 and PBv cells. Pretreatment with LiCl followed by LPS, significantly downregulated IFNα mRNA expression in both PBR3/PBR7 and PBv cells (*P*< 0.001). Notably, in all treatments, IFNα mRNA expression in PBR3/PBR7 was higher than that in PBv. These results suggested that IRF3/IRF7 can enhance IFNα mRNA expression, especially during inflammatory reactions, and illustrated that IRF3/IRF7 exerted an important role in promoting type I interferons production.

The changes in IL-6 mRNA were different from IFNα mRNA, as shown in Figure 3b and Figure 4b. Compared with the control group, LPS increased IL-6 mRNA abundance in PBR3/PBR7 and PBv cells. However, after blocking the TBK1 pathway, LPS still increased IL-6 mRNA expression both in PBR3/PBR7 and PBv cells (*P*< 0.001). In all treatments, IL-6 mRNA expression in PBR3/PBR7 was lower than that in PBv. These results suggested that IRF3/IRF7 can inhibit IL-6 mRNA expression, especially during inflammatory reactions, independent of the TBK1 pathway. These results suggest that IRF3/IRF7 can attenuate the inflammatory response.

### Swine IRF3/IRF7 actives the TLR4 signaling pathway

**Figure 5:**
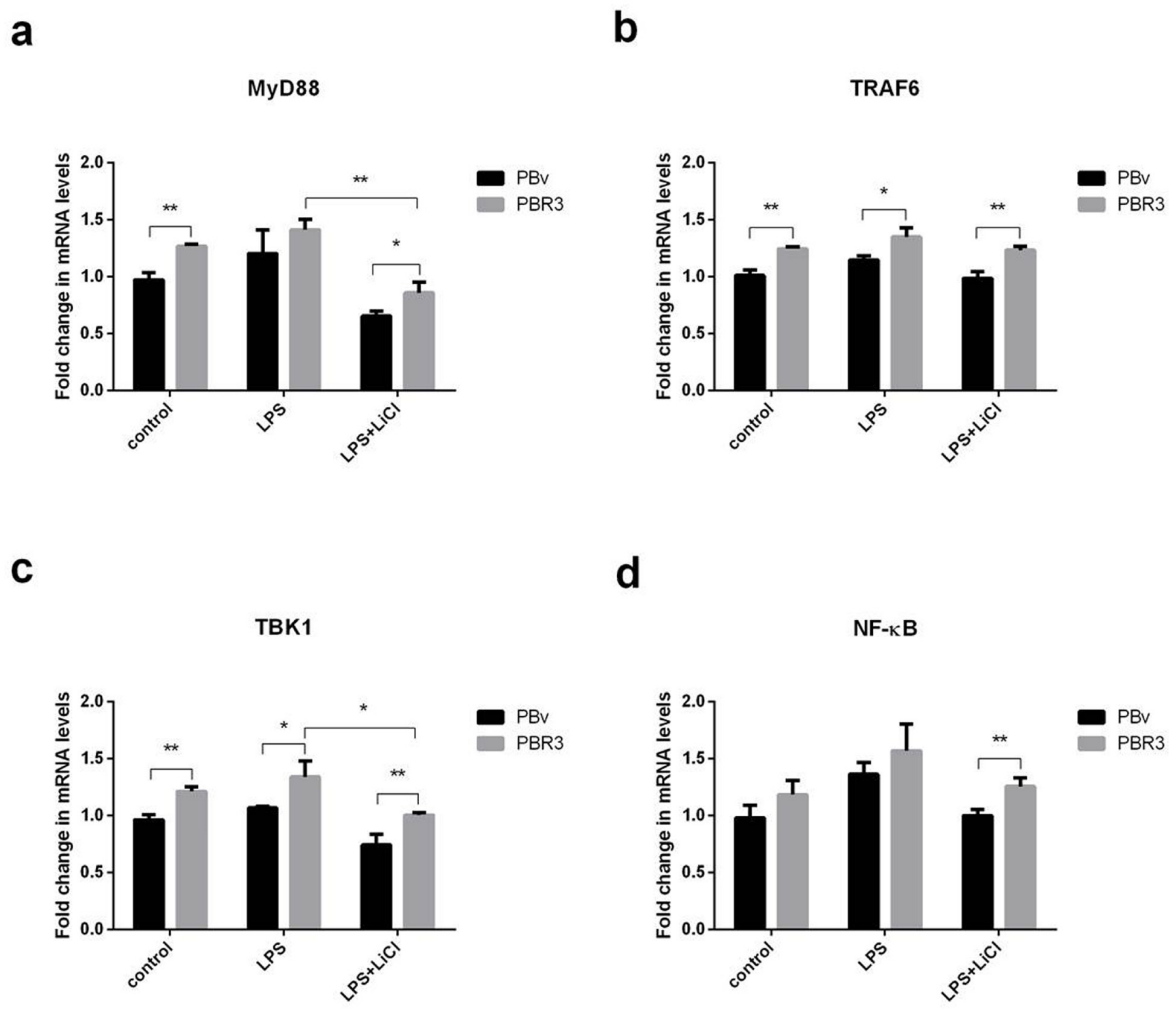
Effect of swine IRF3 overexpression on TLR4 signaling pathway components Graphs demonstrate the expression of MyD88 **(a)**, TRAF6 **(b)**, TBK1 **(c)** and NFκB **(d)** in PBR3 and PBv cells stimulated with LPS (50 μg/ml) with or without LiCl (20 mM) pretreatment. * *P* < 0.05, ** *P* < 0.01 vs. PBv or between treatments.

**Figure 6:**
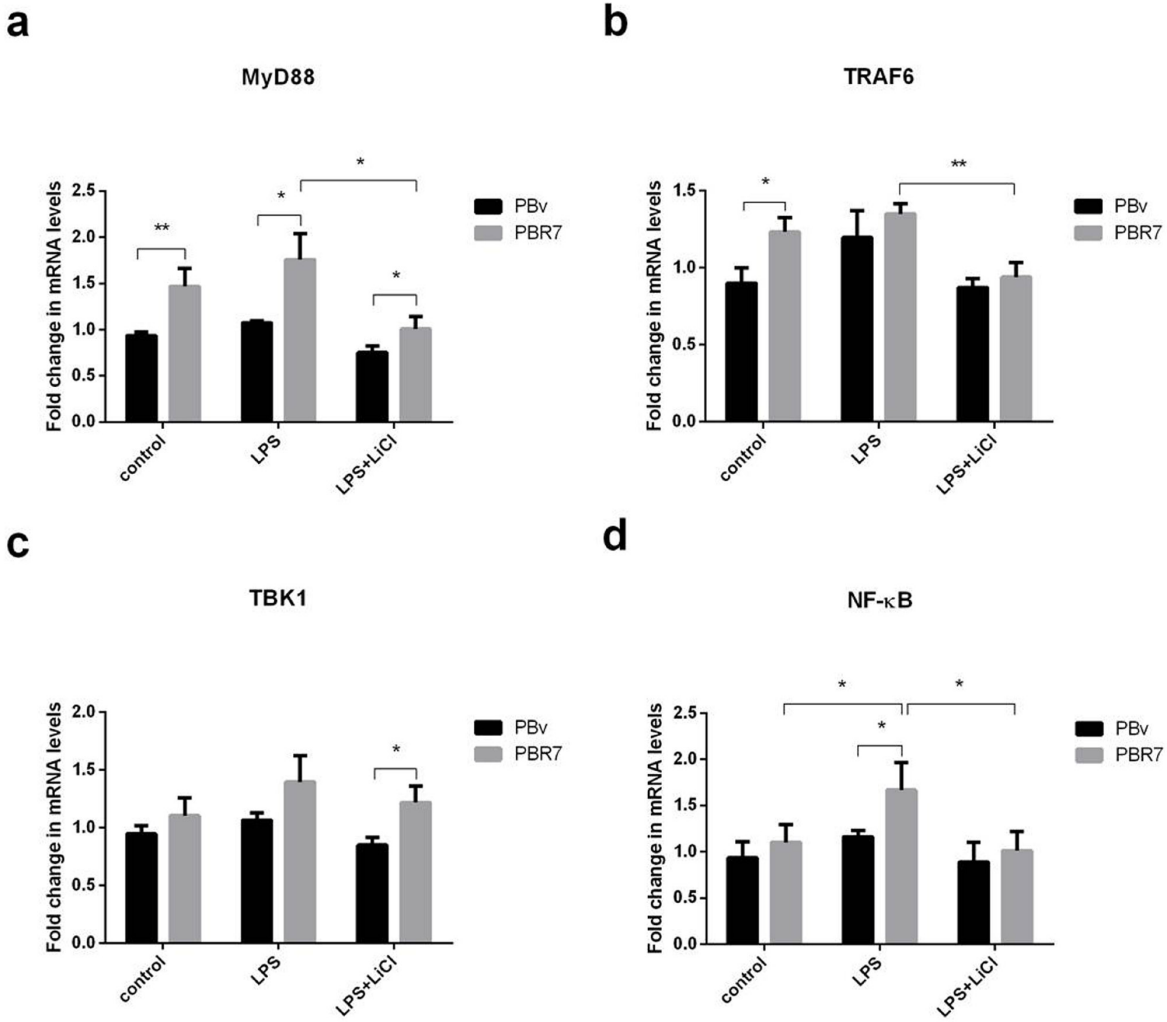
Effect of swine IRF7 on the expression of components of the TLR4 signaling pathway Graphs demonstrate the expression of MyD88 **(a)**, TRAF6 **(b)**, TBK1 **(c)** and NFκB **(d)** in PBR7 and PBv cells stimulated with LPS (50 μg/ml) with or without LiCl (20 mM) pretreatment. * *P* < 0.05, ** *P* < 0.01 vs. PBv or between conditions.

LPS activates multiple TLR4-mediated signal transduction pathways, including a downstream MyD88-independent pathway, leading to IRF3 and IRF7 phosphorylation, which regulates expression and secretion of type I IFNs. Since IRF3 and IRF7 regulate IFNα and IL-6 production, we investigated whether IRF3/IRF7 also have effects on upstream molecules in the TLR4-mediated signaling pathway using real-time PCR. As shown in Figure 5 and Figure 6, we found that changes in MyD88, TRAF6, TBK1 and NFκB mRNA were similar in all treatments. Compared with the control group, LPS increased MyD88, TRAF6, TBK1 and NFκB mRNA levels in PBR3/PBR7 and PBv cells. Pretreatment with LiCl prior to LPS stimulation, downregulated MyD88, TRAF6, TBK1 and NFκB mRNA expression both in PBR3/PBR7 and PBv cells. In all treatments, MyD88, TRAF6, TBK1 and NFκB mRNA expression in PBR3/PBR7 were higher than those in PBv. Our results suggested that swine IRF3/IRF7 could activate TLR4 signaling pathway.

## DISCUSSION

Transposable elements play an important role through “cut-and-paste” of target DNA during genome editing, leading to product insertional mutagenesis or transgenesis [16, 17]. Piggybac (PB) is a DNA transposon which was originally isolated from the genomes of baculoviruses that infect cabbage looper moth *Trichoplusianini* [18]. PB inserts TTAA sequences at target sites, displaying little selectivity for specific position of the genome and a modest superiority for the sensitivity of DNase I region [19, 20]. A small modification of PB would be effective in helping integration into safe sites. Previous studies have found that PB fused to a DNA binding domain remained active, which can distinguish insertion from innate sites [21, 22]. In this experiment, PB transposons as transgene vectors were successfully constructed using the eukaryotic expression vector PB-IRF3 and PB-IRF7, and then the PBase and PB vector plasmid with target gene were co-transfected into PK15 cells. After puromycin-selection, we eliminated non-transfected cells. All cells were clearly observed using fluorescence microscopy after transfection, suggesting that PB-IRF3/IRF7 and the PB-vector were effectively transfected into PK15 cells. From the results of transfection in this study, we found that the efficiency of integration of exogenous genes was affected by the size of DNA fragments, it may be that shorter fragmented genes are more easily integrated into the host. Real-time PCR results showed that IRF3 and IRF7 mRNA expression were obviously higher in PBR3/PBR7 than those in PBv (*P*< 0.001).

IFNs are essential for both innate and adaptive immunity, they play an important role in initially recognizing virus infection and evoking antiviral responses by the immune system. IFN combines with their receptors on the surface of cells, subsequently initiating signaling cascades through Janus kinase-induced signal activators and transducers. The IFN signaling pathway exerts major roles in regulating the expression of various IFN-stimulated genes, which are mainly associated with innate immunity, and thus determine the immune state of the body. Different ligands can bind to TLRs that activate the transcription factors NFκB, IRF3 and IRF7, which are all required for initiating the production of IFNα/β [23]. IRF3/IRF7 is inactive form in the cytoplasm, best known as an important regulator of type I IFN response [24, 25]. When cells are stimulated, IRF3/IRF7 are phosphorylated and change conformation, translocating to the nucleus.

LPS is a principal component of the cell wall in Gram-negative bacteria [26]. LPS recognition and signal transduction are key events in the host immune reaction, and are related to many inflammatory diseases. LPS can activate multiple signal transduction pathways. In the presence of LPS-binding protein (LBP) and CD14, LPS binds with TLR4, recruiting adaptor protein MyD88 and interleukin-1 (IL-1) receptor-associated kinase, followed phosphorylated and activated tumor necrosis factor receptor-associated factor-6 (TRAF6). The activated TRAF6 enhances the activity of the transcription factor NFκB, leading to activation of MAP kinase pathways. Finally, cells produce and release many pro-inflammatory cytokines, such as tumor necrosis factor α (TNFα), IL-6 and IL-8 [27–30]. Lithium salt is a widely used glycogen synthase kinase-3β (GSK3β) inhibitor and an effective drug for treating inflammatory diseases [31, 32]. Furthermore, previous studies have demonstrated that GSK3β is involved in innate immune reactions [33]. For example, GSK3β has been documented as a major regulator maintaining the balance of pro- and anti-inflammatory actions associated with TLR stimulation [34]. Wang et al. demonstrated that lithium decreases IFN-β levels and attenuates antivirus role of host through targeting TBK1 [35].

IRF3 and IRF7 are central players of the signaling cascade leading to IFN type I induction [36]. The activated IRF3 bind to the promoters of IFN type I upregulate IFN-α/β transcription, associating with transcriptional coactivators [37, 38]. Rocca and coworkers showed that the virus induced the inhibition of transcription of the IRF3 gene, decreasing IRF3 translocation to the nucleus furthor inhibits IFN- α/β production [39]. However, Bauthofer et al demonstrated that IRF3 degradation via a proteasome-dependent mechanism, rather than inhibiting the phosphorylation and translocation of IRF3 in classical swine fever virus infected. IRF7 expression, however, is not affected.

In this paper, we explored the effect of overexpression of swine IRF3/IRF7 on IFNα and IL-6 production. Results showed (Figure 3a and Figure 4a) that LPS increased IFNα mRNA abundance with and without overexpression IRF3/7 cells. LiCl significantly downregulated IFNα mRNA expression in both PBR3/PBR7 and PBv (*P*< 0.001). In all treatments, overexpression IRF3/7 increased IFNα mRNA expression. These results suggested that IRF3/IRF7 can enhance IFNα mRNA expression, especially during inflammatory reactions, at the same time also illustrated that IRF3/IRF7 exerted an important role in activating type I interferon response.

IRF3/7 promotes type I IFN transcription in the nucleus [6, 40]. IFNs are secreted binding to specific receptors as an endocrine or autocrine manner, triggering the activation of hundreds of IRFs that participate in antiviral pathways [40, 41]. Recently, Ramírez-Carvajal has demonstrated that a constitutively active fusion protein of porcine IRF3 and IRF7 completely protects swine against foot and mouth disease by inducing a strong type I IFN response [42, 43].

The changes in IL-6 mRNA were different from IFNα mRNA, as shown in Figure 3b and Figure 4b. LPS increased IL-6 mRNA abundance with and without overexpression IRF3/7 cells. After blocking the TBK1 pathway, LPS still increased IL-6 mRNA expression both in PBR3/PBR7 and PBv cells (*P*<0.001). In all treatments, overexpression IRF3/7 decreased IL-6 mRNA. These results suggested that IRF3/IRF7 can inhibit IL-6 mRNA expression, especially during inflammatory reactions independent of the TBK1 pathway. These data suggested that swine IRF3/IRF7 attenuate inflammatory responses. However, Li et al found that IRF7 overexpressing in human microglia increased IL-6 secretion in tissues and serums, promoting signal transducers and activators of transcription 3 (stat3)-IL-6 signaling activation [44]. The conflict conclusion may result from different cell and microenvironment, as well as different detection method.

LPS activates multiple TLR4-mediated signal transduction pathways, activating the downstream MyD88-independent signaling pathway, leading to IRF3/IRF7 phosphorylation, and regulation of the expression and secretion of type I IFNs. Components of swine viruses inhibit IRF3-complex formation in the nucleus [45], which prevents binding to the IFN-β promoter regions and inhibits the assembly of RNA polymerase II of IFN gene expression. In addition, miR-23 induced type I IFN expression through activation of IRF3/IRF7, whichmight further inhibit virus infection [46]. Since IRF3/IRF7 can regulate IFNα and IL-6 production, we investigated whether IRF3/IRF7 has effects on upstream molecules of the TLR4-mediated signaling pathway. As shown in Figure 5 and Figure 6, we found that MyD88, TRAF6, TBK1 and NFκB mRNA variation trends were similar in all treatments. LPS increased MyD88, TRAF6, TBK1 and NFκB mRNA abundance in PBR3/PBR7 and PBv cells. Pretreatment with LiCl significantly downregulated LPS-induced MyD88, TRAF6, TBK1 and NFκB mRNA expression both in PBR3/PBR7 and PBv cells. In all treatments, MyD88, TRAF6, TBK1 and NFκB mRNA expression in PBR3/PBR7 were higher than those in PBv cells. Our results suggested that swine IRF3/IRF7 could activate TLR4 signaling pathway.

## MATERIALS AND METHODS

### Reagents

LPS (*E. coli* O55:B5), puromycin and LiCl were purchased from Sigma (St Louis, MO, USA). Dulbecco’s modified Eagle’s medium (DMEM) and fetal bovine serum (FBS) were obtained from Gibco (Life Technologies, Carlsbad, CA, USA). Trizol reagent, Prime Script RT reagent kit, SYBR Premix Ex Taq, and pMD19-T were purchased from TaKaRa Bio Inc. (Shiga, Japan). PB vector and PB transposes were obtained from SBI Ltd. (Palo Alto, CA, US). Other reagents were purchased from Sino Pharm Chemical Reagent Ltd. (Shanghai, China).

### Plasmids

Construction of the eukaryotic expression vectors PB-IRF3 and PB-IRF7 were as follows. Total RNA was obtained from swine tissues using Trizol reagent, and then cDNA was synthetized using the extracted RNA. IRF3 and IRF7 fragments were amplified and the PCR products were purified using a gel extraction kit. The purified products were ligated into pMD19-T plasmids, and used to transform *E. coli* DH5a cells. Recombinant plasmids were extracted from bacterial colonies, and the presence of IRF3 or IRF7 sequence identified in plasmids by agarose gel electrophoresis. The length of IRF3 and IRF7 sequences were 1260 bp and 1470 bp, respectively. IRF3 and IRF7 cDNA was subcloned from pMD19-T-IRF3 or pMD19-T-IRF7 into the PB vector (that contained green fluorescent protein (GFP) and puromycin resistance genes) after double restriction enzyme digestion. The obtained recombinant expression vectors were called PB-IRF3 and PB-IRF7. *Nhe I* and *EcoR I* were used to digest PB-IRF3 or PB-IRF7, and then identified them through agarose gel electrophoresis. Sequencing using both forward and reverse primers was performed to confirm the above recombinant vectors.

### Cell culture, transfection, and photograph

PK15 porcine kidney epithelial cells were maintained in DMEM medium including 10% FBS (v/v) and 1% penicillin/streptomycin (v/v), and cultured at 37°C with 5% CO_2_ (MCO-5AC CO_2_ Incubator, Sanyo, Tokyo, Japan). Prior to transfection experiments, cells were plated at a density of 2×10^5^ cells per well in six-well plates. The PK15 cells were transfected with PB-IRF3 or PB-IRF7 constructs in parallel with empty vectors using lipofectamine 2000 reagent (Life Technologies) complying with the manufacturer’s instructions. Based on the transfecting plasmids, cells were divided into four groups: PB-IRF3, PB-IRF7, PB-vector and non-transfection. Twelve h after transfection, photomicropgraphs were taken through a DAS microscope (Leitz DM RB) with a charged coupled device camera (C4880; Hamamatsu, Japan) to detect GFP expression. The transfected cells were selected for on 5 μg/mL puromycin for 2 weeks. Cells that were not transfected with the target gene were killed, and we achieved stable transfection cell lines, named PK15-PBIRF3 (PBR3), PK15-PBIRF7 (PBR7), and PK15-PBvector (PBv).

### Transfection efficiency and overexpression detection

The PB vector carries both the purine gene and the GFP gene. Therefore, when the PB vector was transfected into cells and integrated into the genome, GFP protein was expressed in the host cells. After 12 h of transfection, the cells were observed under the fluorescence microscope to evaluate transfection efficiency. Overexpression of target genes (IRF3, IRF7) were detected using RT-PCR.

### Cell treatment

The stably transfected cell lines, PBR3, PBR7, and PBv, were separated into 3 groups: control, LPS, and LPS+LiCl. Cells were exposed to LPS (50 μg/ml) for 6 h in the LPS group. Cells in the LPS + LiCl group were treated with 20 mMLiCl (an inhibitor of TBK1, which inhibits IRF3/7 activity, and interferes with IFN secretion) for 6 h before the addition of LPS (50 μg/ml) for 6 h. Cells in the control group were cultured in basal medium. After treatment, cells were collected to carry out TLR4 signaling pathway and inflammatory markers analysis using real-time qPCR.

### cDNA synthesis and PCR

Trizol reagent was used to extract total RNA from cultured cells. A reverse transcriptase kit was used to carry out cDNA synthesis. PCR analysis was performed with a SYBR Green PCR Kit using a real-time fluorescence quantitative PCR instrument (Eppendorf Mastercycle reprealplex, Hamburg, Germany), with a reaction volume of 20 μL. The PCR program was as follows: 95°C 20 s, 60°C 30 s, and 72°C 20 s, for 40 cycles. Swine mRNA specific primers are listed in Table 1. The synthesis of all primers was carried out by Shanghai Sangon Co. Ltd. (Shanghai, China). Each sample was analyzed in triplicate, and RT-PCR results were analyzed and evaluated using the relative quantity Ct method. Melting curve analysis was used to confirm the specificity of primers. The expression of the target gene was normalized as the ratio of target gene/GAPDH mRNA.

**Table 1:** Primers used for RT-PCR amplification

Gene	Primers	Accession No.
IRF3	F: 5’-TCATCGAAGATCTGATTGCCTTC-3’ R: 5’-GGGACAACCTTGACCATCACC-3’	NM_213770.1
NFκB	F: 5’-CCCATGTAGACAGCACCACCTATGAT-3’ R: 5’-ACAGAGGCTCAAAGTTCTCCACCA-3’	NM_001048232.1
IRF7	F: 5’-CTGCGATGGCTGGATGAA-3’ R: 5’-TAAAGATGCGCGAGTCGGA-3’	NM_001097428.1
MyD88	F: 5’-GCTGTAGGGGGAATGTGTGT-3’ R: 5’-GGCTCTGGTTTCCACTGTCC-3’	XM_013992286.1
TRAF6	F: 5’-GGGAACGATACGCCTTACAA-3’ R: 5’-CTCTGTCTTAGGGCGTCCAG-3’	NM_001105286.1
IL-6	F: 5’-CTGGCAGAAAACAACCTGAACC-3’ R: 5’-TGATTCTCATCAAGCAGGTCTCC-3’	NM_001252429.1
TBK1	F: 5’-ACAGATTTTGGTGCAGCCAG-3’ R: 5’-CCTTATTCCTACGTGGCCCT-3’	NM_001105292.1
IFNα	F: 5’-GGTGCATGAGATGCTCCA-3’ R: 5’-GCCGAGCCCTCTGTGCT-3’	XM_013993516.1
TLR4	F: 5’-CCCTGACAACATCCCCACAT-3’ R: 5’-AAAGGCTCCCAGGGCTAAAC-3’	XM_013986843.1
GAPDH	F: 5’-GGAGAACGGGAAGCTTGTCA-3’ R: 5’-GCCTTCTCCATGGTCGTGAA-3’	NM_001206359.1

### Statistical analysis

All results are presented as the mean ± standard deviation. SPSS (Statistical Package for the Social Sciences) statistical software (version 13.0) was used to analyze data (IBM SPSS Inc., Armonk, NY, USA). One-way analysis of variance (ANOVA) was employed to analyze the differences among groups. P-values of *P*< 0.05 were taken to indicate statistically significant differences.

## Abbreviations

LPS: lipopolysaccharide
LiCl: lithium chloride
PRRS: porcine reproductive and respiratory syndrome
PRRSV: porcine reproductive and respiratory syndrome virus
IFNs: Type I interferons
TLRs: Toll-like receptors
IRFs: Type I interferons regulatory factors
PB: Piggyback
LBP: LPS-binding protein
MyD88: myeloid differentiation factors
IL-1: interleukin-1
TNFα: tumor necrosis factor α
GSK3β: glycogen synthase kinase-3β
DMEM: Dulbecco’s modified Eagle’s medium
FBS: fetal bovine serum
GFP: green fluorescent protein
PBR3: PK15-PBIRF3
PBR7: PK15-PBIRF7
PBv: PK15-PBvector
ANOVA: one-way analysis of variance
TRAF6: tumor necrosis factor receptor-associated factor-6
stat3: signal transducers and activators of transcription 3
